# PEPFAR support of alcohol–HIV prevention activities in Namibia and Botswana: a framework for investigation, implementation and evaluation

**DOI:** 10.1017/gmh.2015.24

**Published:** 2016-01-26

**Authors:** M. Glenshaw, N. Deluca, R. Adams, C. Parry, K. Fritz, V. Du Preez, K. Voetsch, P. Lekone, P. Seth, P. Bachanas, M. Grillo, T. F. Kresina, B. Pick, C. Ryan, N. Bock

**Affiliations:** 1 Division of Global HIV/AIDS, Center for Global Health, Centers for Disease Control and Prevention, Pretoria, South Africa; 2 Division of HIV/AIDS Prevention, National Center for HIV/AIDS, Viral Hepatitis, TB Prevention, Centers for Disease Control and Prevention, Atlanta, GA, USA; 3 Ministry of Health and Social Services, Windhoek, Namibia; 4 Alcohol, Tobacco & Other Drug Research Unit, Medical Research Council, Cape Town, South Africa; 5 Department of Psychiatry, Stellenbosch University, Cape Town, South Africa; 6 International Center for Research for Women, Washington, DC, USA; 7 Division of Population Health, National Center for Chronic Disease Prevention and Health Promotion, Centers for Disease Control and Prevention, Atlanta, GA, USA; 8 Division of Global HIV/AIDS, Center for Global Health, Centers for Disease Control and Prevention, Gaborone, Botswana; 9 Division of Global HIV/AIDS, Center for Global Health, Centers for Disease Control and Prevention, Atlanta, GA, USA; 10 US Department of Defense, San Diego, CA, USA; 11 Division of Pharmacologic Therapies, Center for Substance Abuse Treatment, Substance Abuse and Mental Health Services Administration, Rockville, MA, USA; 12 United States Agency for International Development, Washington, DC, USA

**Keywords:** Alcohol, HIV, PEPFAR, Namibia, Botswana

## Abstract

**Background:**

The association between harmful use of alcohol and HIV infection is well documented. To address this dual epidemic, the US President's Emergency Plan for AIDS Relief (PEPFAR) developed and implemented a multi-pronged approach primarily in Namibia and Botswana. We present the approach and preliminary results of the public health investigative and programmatic activities designed, initiated and supported by PEPFAR to combat the harmful use of alcohol and its association as a driver of HIV morbidity and mortality from 2008 to 2013.

**Approach:**

PEPFAR supported comprehensive alcohol programming using a matrix model approach that combined the socio-ecological framework and the Alcohol Misuse Prevention and Intervention Continuum. This structure enabled seven component objectives: (1) to quantify harmful use of alcohol through rapid assessments; (2) to develop and evaluate alcohol-based interventions; (3) to promote screening programs and alcohol abuse resource services; (4) to support stakeholder networks; (5) to support policy interventions and (6) structural interventions; and (7) to institutionalize universal prevention messages.

**Discussion:**

Targeted PEPFAR support for alcohol activities resulted in several projects to address harmful alcohol use and HIV. Components are graphically conceptualized within the matrix model, demonstrating the intersections between primary, secondary and tertiary prevention activities and individual, interpersonal, community, and societal factors. Key initiative successes included leveraging alcohol harm prevention activities that enabled projects to be piloted in healthcare settings, schools, communities, and alcohol outlets. Primary challenges included the complexity of multi-sectorial programming, varying degrees of political will, and difficulties monitoring outcomes over the short duration of the program.

## Introduction

The harmful use of alcohol, defined by WHO as alcohol consumption with adverse health and social outcomes at both the individual and societal level, is prevalent in sub-Saharan Africa and associated with excessive burden of disease and injury (WHO, 2014*c*). When coupled with the highest global HIV burden, sub-Saharan Africa faces dual and connected epidemics that substantially threaten morbidity, mortality, and productivity. This paper describes the HIV and alcohol use burdens in Southern Africa, specifically Namibia and Botswana, and outlines research, program, and policy actions undertaken by national governments and implementers with the support of the US President's Emergency Plan for AIDS Relief (PEPFAR) to address alcohol-related HIV issues.

## Background

Several studies in sub-Saharan Africa have demonstrated the association between both all levels of alcohol use and HIV infection (Fritz *et al*. 2002, 2010; Fisher *et al*. 2007, 2008; Kalichman *et al*. 2007; Baliunas *et al*. 2010; Shuper *et al*. 2010). Alcohol-associated HIV transmission is described in a meta-analysis, demonstrating higher risk of incident HIV infection among drinkers compared with non-drinkers [relative risk (RR) = 1.77, 95% confidence interval (CI) 1.43–2.19], even higher risk for persons drinking alcohol before or during sex (RR = 1.87, 95% CI 1.39–2.5), and highest risk for heavy episodic, or binge, drinking (RR = 2.2, 95% CI 1.29–3.74) (Baliunas *et al*. 2010).

Alcohol use can reduce an individual's ability to learn and perform sexual risk reduction strategies (Kalichman *et al*. 2007). Multiple studies have found that any use of alcohol in sexual situations is associated with lower condom use, increased casual sexual encounters, greater numbers of sex partners, higher risk sex partners, and greater multiple concurrent partnerships (Fritz *et al*. 2002; Maisto *et al*. 2004; Lewis *et al*. 2005). Drinking establishments have been reported to be venues for meeting casual and transactional sex partners, with 85% of patrons reporting meeting new sex partners in bars, and 45–57% of bar patrons reporting multiple concurrent sexual partnerships in South Africa and Zimbabwe (Lewis *et al*. 2005; Morojele *et al*. 2006; Kalichman *et al*. 2008). In addition, harmful use of alcohol also plays a critical role in excessive morbidity among persons living with HIV, particularly non-adherence to antiretroviral treatment (Crosby *et al*. 2008; Kalichman *et al*. 2008).

Estimated rates of alcohol consumption among drinkers in the WHO African Region are second highest in the world at 19.5 L of pure alcohol per capita, and heavy episodic (i.e. binge) drinking prevalence in the African region is third highest globally at 16.4% (WHO, 2014*c*). The WHO Global Status Report on Alcohol Use in 2014 noted that southern African countries, including Namibia and Botswana, had particularly high rates of heavy episodic drinking despite high levels of alcohol abstinence among the general population (WHO, 2014*c*). In Namibia, while 50.8% of men and 70.2% of women abstained from alcohol consumption in the past year, over 37.2% of adult drinkers reported heavy episodic drinking at least monthly, with high binge drinking prevalence among both men (46.4%) and women (23.6%) (WHO, 2014*b*). High prevalence of alcohol use among teens and young adults has also been reported in Namibia, with 33% of 13–15 year olds reporting current alcohol use and over one-third of young drinkers reporting having been drunk in their lifetime (WHO Regional Office for Africa, 2008). In Botswana, similar high rates of alcohol abstention exist (43.6% among men and 73.5% among women), with greater sex differences in binge drinking prevalence (17.2% overall, 22.6% among men, and 5.8% among women) (WHO, 2014*a*). Both Namibia and Botswana have substantial adult HIV prevalence in relatively small populations; Namibia's overall HIV prevalence in 2013 was estimated to be 14.3% (95% CI 11.8–17.3) in a population of 2.3 million, while Botswana's overall HIV prevalence was estimated to be 21.9% (95% CI 20.8–23.1) in a population 2.0 million (UNAIDS, 2013*a*, *b*).

Despite the known harms of the harmful use of alcohol and its strong association with HIV, there are currently limited surveillance activities to record alcohol production, consumption, harm, and its health impact in sub-Saharan Africa generally. Thus, a regional strategy on reduction of the harmful use of alcohol was endorsed by the Regional Committee for Africa in 2010 (Resolution AFR/RC60/R2) (WHO, 2014*c*). Given the prevalence of alcohol consumption in these high HIV-burden settings, there is considerable opportunity for alcohol-related HIV transmission. These countries have developing public health systems and services that address most basic health needs, such as primary care and maternal/child health, but have been significantly burdened by the HIV epidemic of the past two decades. Health issues directly and indirectly influenced by harmful use of alcohol are generally considered of secondary importance in these settings. Alcohol-related programming challenges are magnified with the popularity of production and consumption of homebrewed alcohol, which compound alcohol measurement and control activities.

The dual epidemics of harmful use of alcohol and high HIV prevalence in sub-Saharan Africa have substantial implications for public health programs and donor investments. In 2007, in response to growing evidence of the harmful use of alcohol as a driver of the HIV epidemic, PEPFAR allocated special funding to address harmful alcohol use as a HIV prevention strategy. This paper presents the approach and preliminary results of the public health investigative and programmatic activities initiated and supported by PEPFAR to combat the harmful use of alcohol and its association as a driver of HIV morbidity and mortality in sub-Saharan Africa from 2008 to 2013. While not a research article, activities and outcomes are presented in a systematic fashion to contribute to the growing theoretical, methodological, and programmatic knowledge of the emerging field of alcohol and HIV.

## Approach

The interagency PEPFAR alcohol initiative was led by the Centers for Disease Control and Prevention (CDC) and involved the US Agency for International Development (USAID), the US Department of Defense (DOD) and the US Substance Abuse and Mental Health Services Administration (SAMHSA). The initiative was developed through consultation with a wide variety of subject matter experts, including researchers, addiction specialists, and program experts across multiple sectors. Consultations occurred outside of the health sector, with law enforcement, media, trade and industry, and education specialists, in addition to the more traditional health stakeholders, including injury, gender, mental health, and chronic health experts. The results of these consultations and interagency approach culminated in a matrix approach to conceptualize and execute a variety of activities in a comprehensive manner.

**Fig. 1.**
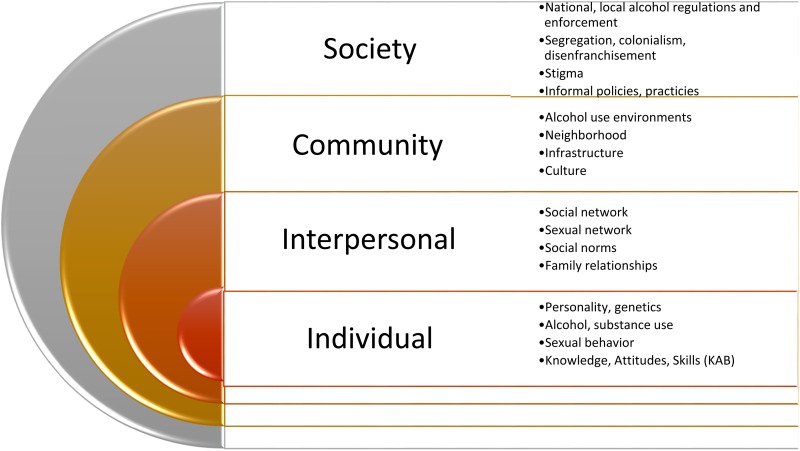
Socio Ecological Framework of Alcohol-Related HIV Risk (Adapted from Scribner *et al*. 2010).

**Fig. 2.**
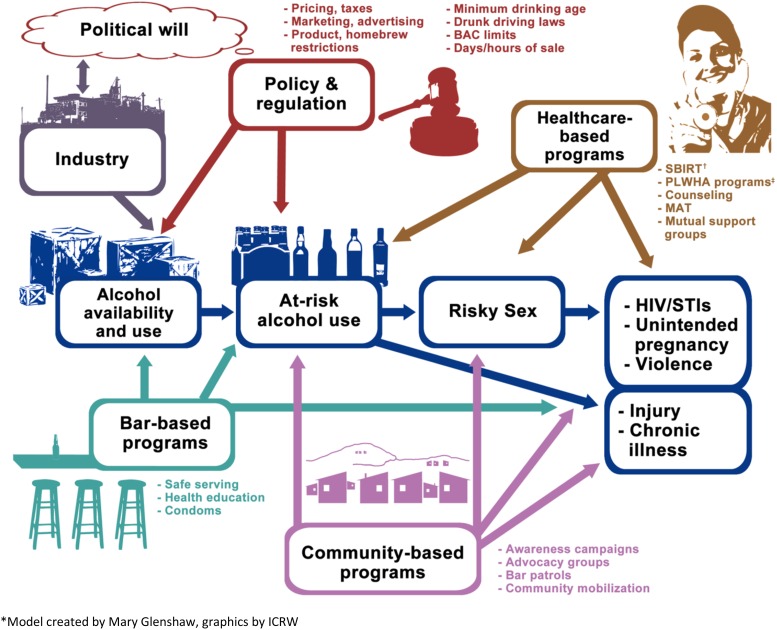
Alcohol misuse prevention and intervention continuum*. *Model created by Mary Glenshaw, graphics by ICRW. ^†^Screening, Brief Intervention, Referral to Treatment (SBIRT). ^‡^Persons Living with HIV/AIDS (PLWHA).

Following subject matter consultations, the interagency PEPFAR team developed a comprehensive alcohol–HIV prevention approach using a three-step process. The theoretical basis of the initiative (Step 1) involved the socio-ecological framework (Fig. 1, Scribner *et al*. 2010) to encompass the complexity of individual, interpersonal, community, and societal factors that influence the harmful use of alcohol. In Step 2, we designed a graphic depiction of intervention points, termed the Alcohol Misuse Prevention and Intervention Continuum (Fig. 2), which includes upstream primary prevention approaches such as restrictions on alcohol availability; secondary prevention, such as structural interventions to minimize opportunities for the harmful use of alcohol; and tertiary prevention to reduce the health impacts of alcohol, such as screening and intervention programs. Finally, Step 3 involved the merging of the socio-ecological framework with the prevention continuum into a matrix model to depict objectives and activities to optimally measure, reduce, and prevent alcohol-related harms.

**Fig. 3.**
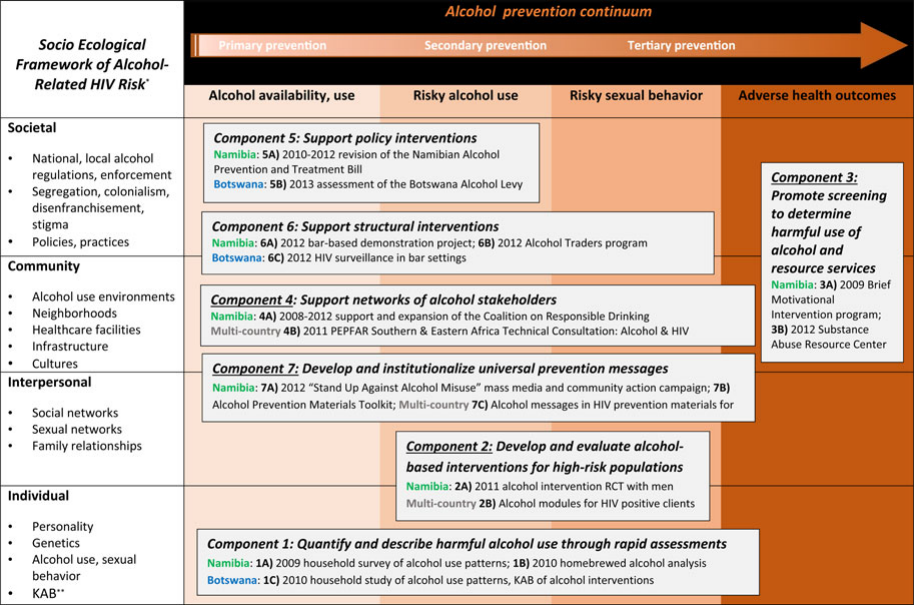
Investigative and programmatic matrix model of the PEPFAR Alcohol Initiative, 2009–2013. *Adapted from Scribner *et al*. (2010). ** Knowledge, Attitudes, Behaviors (KAB).

This matrix model (Fig. 3) enabled articulation of health policy, behavioral science, epidemiology, addiction treatment, and development interventions across six component objectives: (1) to quantify and describe harmful use of alcohol through rapid assessments to better understand the local context; (2) to develop and evaluate alcohol-based interventions for high-risk populations to prevent HIV acquisition, transmission and comorbidities, (3) to promote screening to detect harmful use of alcohol and promote the use of alcohol abuse resource services, particularly in healthcare settings, (4) to support networks of stakeholders to better leverage the multi-sectorial interests and engagement in alcohol programming; (5) to support policy interventions and (6) structural interventions to prevent harmful use of alcohol using upstream approaches; and (7) to institutionalize universal prevention messages to increase and standardize consistent communication regarding the reduction of harmful alcohol use. Figure 3 depicts these objectives across the matrix, with the risk levels conveyed by the prevention continuum (x axis) and the factors depicted by the socio-ecological framework (y axis), along with highlighted activities implemented in Namibia, Botswana and elsewhere in sub-Saharan Africa. We focused the majority of activities in Namibia because of demonstrated need in terms of both high HIV burden and alcohol use, along with existence of a passionate but under-resourced Alcohol Unit in the Namibian Ministry of Health and Social Services. Fewer activities were implemented in Botswana for several reasons, including better resources overall, strong anti-alcohol advocacy in the Office of the President, and rapid progress toward strong alcohol control legislation. As a result, activities were not mirrored in both countries, and were rather tailored to each country's local need and context. Thus, this paper does not aim to compare activities or results between countries; rather, we present approaches and findings undertaken through the PEPFAR program, using the matrix model described.

## Component 1: Rapid assessments

The quantification and description of harmful patterns of alcohol use is a crucial cornerstone to primarily understanding the context of alcohol consumption in limited resource settings. Following the matrix model described (Fig. 3), this first component spans all levels of the risk continuum through the assessment of alcohol use, harmful use, associated risky sexual behavior, and associated adverse health outcomes. Such assessments generally occur at the individual level in the socio-ecological framework. As neither the national alcohol program in Namibia nor in Botswana have routine alcohol use surveillance in place, PEPFAR supported a variety of rapid assessments in both countries. Together with host government counterparts and local partners, the PEPFAR team aimed to quantify and contextualize the populations affected and those at risk of harmful alcohol use, as well as consumption patterns and alcohol types. The critical basic steps of these assessments involved determination of gaps and priorities through a background review of activities, literature, local legislation and policies, as well as discussions with existing stakeholders vested in alcohol harm reduction, followed by the design and execution of simple, focused assessments.

In Namibia, through consultation with stakeholder networks and local champions, we determined that developing appropriate alcohol intervention programming among populations at high risk of HIV required better understanding of the patterns of drinking and types of alcohol consumption in a highly impoverished urban community. In 2009, the PEPFAR team worked with the nongovernmental organization Human People-to-People and the Ministry of Health and Social Services, to conduct a population-based survey of drinking behaviors in the former township, Katutura, outside of Windhoek, the Namibian capital. Katutura was established for non-white residents during the Apartheid era when Namibia was under the jurisdiction of the South African government and includes a diverse ethnic population estimated between 100 000 and 200 000 persons, including large communities living in informal settlements without electricity, piped sewage, or water systems. In Botswana, we worked with University Research Council in 2010 to conduct a rapid situational assessment of alcohol use, as well as quantify local alcohol availability, investigate locally available alcohol abuse interventions and services, and better understand perceptions of recent national alcohol regulations. This rapid assessment involved a household study conducted in Palapye, a large urban village and in Letlhakeng, a smaller, more rural community using a mixed quantitative and qualitative approach of survey methodology, focus group discussions and in-depth interviews.

The methods, key overall findings, key binge drinking-related findings, and reporting of homebrew and commercial alcohol from both studies are summarized in Table 1. In addition to these key alcohol use findings, we further estimated that in Namibia, proportions of daily, weekly, and monthly binge drinking behaviors of men were estimated to be double those of women, and while proportions of high-intensity binge drinking (≥10 drinks) with high frequency (daily or almost daily) were high for both sexes, men reported engaging in this behavior nearly four times more than women (16.2% *v*. 4.8%, *p* < 0.05) (Seth *et al*. 2015). Further, women who made or sold alcohol from their homes were more likely to report hazardous or harmful drinking patterns compared with women who were not involved with alcohol production or sales, after controlling for age and employment status (AOR = 1.8, 95% CI 1.1–3.2) (Seth *et al*. 2015).

**Table 1.** Summary of rapid assessments to contextualize alcohol use in Namibia and Botswana, 2011

Country, year	Methodology, sample size	Key findings^a^		
		Overall	Binge drinking^c^	Homebrew, commercial alcohol
Namibia, 2009	• Quantitative individual survey, including AUDIT^b^ and home alcohol production factors • Multi-stage stratified sampling of 25 000 households in urban township • 400 households, 639 individuals	• 73% estimated current alcohol use • 44% estimated hazardous and harmful alcohol use^b^ • 11% estimated dependence	• 76% reported consumption of ≥3 drinks daily • 49% reported binge drinking in the previous year • 31% reported binge drinking at least monthly • 16.2% (men), 4.8% (women) reported binge 10+ drinks daily or almost daily	• 35% made and/or sold alcohol from their homes • 79.6% (men), 70.2% (women) reported consuming homebrewed alcohol • 43% reported drinking commercial bottled beer • 92% of alcohol consumption and purchases occurred in home-based bars or *shebeens*
Botswana, 2010	• Mixed methods survey, focus groups, in-depth interviews • Stratified sampling of households, random selection of individuals in urban village, and rural community • 711 individuals	• 33% reported current alcohol use	• 40% reported binge 10+ drinks 2–3*x*/month	• 41% reported drinking Chibuku, a local commercialized traditional beer • 29% reported drinking commercial bottled beer • 18% reported drinking homebrewed beer

a Previous 12 months.

b Alcohol Use Disorders Identification Test (AUDIT) screening tool (Babor *et al*. 2001).

c Findings among current drinkers.

To further investigate homebrewed alcohol in Namibia, we conducted a rapid assessment of the alcohol content of homebrew from a variety of drinks conveniently selected from the same Katutura communities we had surveyed. Laboratory testing of 20 samples of nine varieties of homebrew sampled from home-based shebeens was performed by Namibia Breweries Ltd in 2010. The Alcohol by Volume (ABV) results of the nine varieties ranged from 0.0 to 40.6%, with a median and mean ABV of 3.5% and 6.6%, respectively. The most commonly consumed homebrew in Namibia, ‘Tombo,’ had a median ABV of 4.4% (range 3.5–5.9%) from six different samples. Despite sampling the homebrew from a variety of shebeens scattered over a wide geographic radius, the ABV results for each type was very tightly clustered (Glenshaw *et al*. 2011).

Additional findings from the Botswana assessment revealed survey respondents' perceptions of the impact of various governmental alcohol regulations. Over 72% of all respondents disagreed that the national Botswana alcohol levy, a point of sale tax introduced in 2008, was effective in lowering alcohol consumption. In addition, the majority of respondents disagreed that reduction in alcohol trading hours and the minimum age of purchase at age 18, as mandated by the Liquor Act of 2003, were effective approaches to reducing alcohol use. However, 58% of respondents did agree that new, stricter penalties for drunken driving were an effective deterrent for harmful use of alcohol. While there was equity between sexes and communities for most responses regarding governmental interventions, significantly more male respondents (36.9%) strongly agreed with drunken driving regulation than female respondents (28.4%, *p* < 0.05). The most popular approaches suggested to curb harmful alcohol use in Botswana included complete alcohol prohibition (45.2%) and using harm reduction interventions, such as counseling services, to help drinkers reduce alcohol intake (20.9%). Less than 10% of respondents agreed with imposing a further levy on alcohol purchases, limiting alcohol sales, further reducing trading hours, legalizing informal drinking venues, or raising the legal drinking age to 21 years.

Interviews and focus group discussions in Botswana revealed a mixed perception of harmful alcohol use by participants, and allowed for some examination of influences beyond the individual level in the socio-ecological framework. Behavioral issues on the part of the alcohol consumer, community issues, and alcohol commodity issues emerged. Themes regarding suggestions to curb alcohol consumption from an interpersonal perspective included improving public education and alcohol abuse prevention education in schools. Community and societal themes included improving employment rates and increasing recreational opportunities to reduce drinking associated with idleness, as well as establish rehabilitation and stigma reducing programs to assist persons with alcohol use disorders. Some participants also advocated for structural approaches such as limiting alcohol content in beverages to reduce extreme intoxication. Respondents in both communities in Botswana reported a general lack of services available for persons with alcohol dependence or substance abuse problems. While some HIV support groups were acknowledged to provide support for individuals abusing alcohol, there was a general consensus that formal services were absent and that community groups lacked skills, training, resources, and materials to adequately address alcohol abuse and dependence (University Research Council, 2010).

## Component 2: Interventions and evaluations

The second component objective of the PEPFAR alcohol initiative, the development and evaluation of alcohol-based interventions for high-risk populations, spans the last three areas of the prevention continuum (high-risk alcohol use, high-risk sexual behavior, and adverse health outcomes) at the interpersonal level of the socio-ecological framework, as depicted in Fig. 3. The primary PEPFAR activity in this component was the design, development, implementation, and evaluation of an intervention for men at high risk of harmful alcohol use and HIV acquisition. Given the time and financial resources to undertake this intensive activity, only one country could be selected. Because of the demonstrated high rate of frequent, heavy episodic drinking among men in Katurura documented by the 2009 rapid assessment (16.2% daily or almost daily consumption of 10+drinks), the PEPFAR team selected Namibia as the recipient of this activity.

This intervention was specifically contextualized to the drinking behaviors of men in Namibia. The CDC team used cognitive behavioral theory and counseling techniques based on the Information–Motivation–Behavior change (IMB) model and Motivational Interviewing to design a 1-h, interactive counseling intervention. The intervention built on similar interventions designed by Kalichman, Simbayi and colleagues in South Africa (Simbayi *et al*. 2004) and incorporated other interventions using IMB methods, and interventions promoted through the diffusion of effective behavioraliInterventions (DEBI) service (CDC, 2014). The intervention consists of an individual counseling session with various interactive educational and risk-reduction techniques and activities relating to alcohol consumption and HIV sexual risk behaviors. The intervention components were extensively field tested in Katutura and modified prior to implementation, and included versions in the three most common languages of the area. Counselors were trained to provide a confidential, supportive atmosphere for open communication on risk-reduction and behavior change, and assess alcohol-related sexual risk behaviors through the use of motivational interviewing techniques and the assistance of a scripted job-aid. The CDC team conducted a randomized controlled trial to assess the efficacy of the intervention in reducing alcohol use and risky sexual behavior over 6 months with 550 HIV-negative men in Namibia with AUDIT scores of 8 to 19 (hazardous or harmful alcohol use) and at least two sexual partners. Results of the study will be disseminated in 2016.

PEPFAR also developed an alcohol module for the ‘Prevention with Positives’ (later known as Positive Health, Prevention, and Dignity) program to reduce alcohol use and related complications for persons living with HIV (PLHIV). In preparation for this program in Namibia, CDC investigated alcohol use patterns of a cohort of 1186 PLHIV enrolled in clinical care from 2009 to 2010. Overall, 104 (8.8%) of PLHIV in the Namibia study reported binge drinking six or more drinks in the previous six months, a significantly higher finding than PLHIV counterparts in Kenya (5.4%) and Tanzania (2.2%) (Medley *et al*. 2014). When controlling for other confounders, factors across countries associated with PLHIV engaging in binge drinking included male sex, recent HIV diagnosis, and sexual risk taking, including multiple partners and inconsistent condom use. These findings highlight the need for specific interventions to screen for and address harmful alcohol use in clinical HIV settings. Thus, the Prevention with Positives alcohol module was designed to promote accurate screening of alcohol consumption patterns in HIV clinical settings and address the specific psychosocial and medical issues associated with drinking when living with HIV. Intended outcomes of the program were improved anti-retroviral treatment (ART) adherence and reduced sexual risk behaviors associated with onward HIV transmission; further results from this intervention will be disseminated when available.

Similar activities were also supported by PEPFAR through the DOD using a brief screening tool and an IMB model to support ART adherence among PLHIV using military health facilities in selected countries in sub-Saharan Africa. Unlike the activities for the general population previously described, programmatic uptake and impact results of military-based programs are generally not available publicly given security concerns. However, the PEPFAR DOD team was able to leverage general population findings to customize programs for military populations, an example of the collaborative programming efforts by PEPFAR agencies and host countries to address harmful alcohol use and improve health outcomes.

## Component 3: Promotion of alcohol screening and resource services

The third component of PEPFAR alcohol activities, promotion of alcohol screening and resource services, generally addresses adverse health outcomes within the prevention continuum, and encompasses both societal and community-level approaches in the socio-ecological framework. Highlighted PEPFAR activities were primarily implemented in Namibia to complete the comprehensive programmatic package following identification of harm and application of interventions. Activities included training and piloting of an alcohol screening activity among healthcare providers in the Hardap region of Namibia in 2009, through the Addiction Technology Transfer Center (ATTC) at the University of Texas (Glenshaw *et al*. 2011). This activity involved training in brief motivational interventions (BMI) adapted to include the specific language and terminology, cultural norms, and drinking behaviors unique to the Namibian context. Implementation of the program following training proved difficult for healthcare facilities for several reasons. Despite local context adaptations, the BMI concept was not well understood by many healthcare practitioners. Providers reported that the ‘brief’ aspect of the program (5–10 min) did not allow enough time to build rapport and provide a safe environment for clients to disclose harmful drinking behaviors. In addition to the time trainees preferred to build such a rapport (>30 min), other barriers include perceived stigma, social desirability bias by clients, complexity of assessing drinking levels given the prevalence of consuming homebrewed alcohol and the habit of drink sharing, and the disruption of routine clinical flow and roles in public clinics and hospitals. Following the identification of barriers to implementation of the BMI program, the PEPFAR team supported a BMI program revision that included new components to create more awareness and sensitization of alcohol abuse issues in clinics, emphasize brief assessment and intervention by nursing staff, and allow for referral for alcohol treatment and support to staff better trained to handle psychosocial issues, such as social workers. This revision included a comprehensive set of training and practice materials finalized in 2012, including culturally adapted screening tools and videos of clinical role plays for use by clinical and program partners.

The PEPFAR team also worked closely with Alcoholics Anonymous (AA) and the Etegameno Rehabilitation and Resource Centre to better understand the needs and gaps in addiction services in Namibia from 2008 to 2012. These two organizations were among the few no-cost rehabilitation services available in Namibia, and national resources for expansion, networking, and information were scarce. Stakeholders from these and other groups clearly articulated the need for a center to serve as a safe space for persons recovering from addiction, for family support, information and education, and for space to connect disparate parties working in alcohol abuse prevention. Thus, PEPFAR supported the creation and launch of the Namibian Substance Abuse Resource Center in Katutura in 2012. The center was co-funded by PEPFAR and the Namibian Government, with in-kind renovation assistance from AA and other volunteers, and involved re-purposing a warehouse into an alcohol resource center to provide resources, literature, recreation, vocational skill training, and meeting space for mutual support groups.

## Component 4: Network support

The fourth component objective of the initiative, support of alcohol stakeholder networks, spans the first three areas of the prevention continuum similar to the rapid assessment objective, but addresses community-level factors rather than individual factors in the socio-ecological framework. This component was one of the deciding factors in selecting Namibia as the recipient of most of the support of special PEPFAR alcohol funding and technical assistance (Glenshaw *et al*. 2010). In Namibia, the Coalition on Responsible Drinking (CORD), had formed prior to PEPFAR involvement, and comprised a multi-sectorial network of individuals and organizations interested in curbing harmful alcohol use. PEPFAR supported the expansion and coordination functions of CORD at a national level from 2008 to 2012. This involved strengthening staffing and structure to include a full-time social worker and administrator to better coordinate CORD members and partners, including national and local government, donors, NGOS, and private industry. CORD activities PEPFAR supported included raising awareness through door-to-door campaigns, marches, workshops, recognition days, and radio discussions. CORD also conducted school programs, neighborhood patrols, community clean-up campaigns, and information and referral for alcohol dependence support groups. With PEPFAR support, technical assistance, funding, and monitoring and evaluation guidance, CORD successfully expanded to all 13 regions in Namibia by 2011 and continues to be active (Adams, 2015, personal communication). The national CORD office developed Standard Operating Procedures for regional and local chapters, conducted launches and rallies to expand its reach, and trained social workers to mentor CORD committees throughout Namibia, so that committees became the conduit of all alcohol activities in their jurisdictions. A formal evaluation of the CORD expansion has not been conducted to date, but the MOHSS reports that CORD activities have been sustained in most regions after the conclusion of PEPFAR technical and financial assistance (Adams, 2015, personal communication).

In addition to national network support, we also held the PEPFAR Southern & Eastern Africa Technical Consultation on Alcohol and HIV in Namibia in 2011 with over 80 leading alcohol prevention researchers, programmers, advocates and public officials from 14 countries, including Botswana (Fritz *et al*. 2012). This meeting allowed for exchanges of recent evidence, approaches, intervention ideas and best practices, as well as an opportunity to review WHO and PEPFAR guidance on the prevention of the harmful use of alcohol. Participants engaged in strategic planning workshops to help translate guidance and approaches shared to their local context and continued networking through the PEPFAR Key Populations Technical Working Group following the conclusion of the meeting.

## Component 5: Policy interventions

The fifth component of the PEPFAR initiative was to support one of the most upstream prevention approaches, policy interventions to reduce alcohol availability and decrease harmful use. This component relates the most proximal levels of the prevention continuum and the societal level of the socio-ecological framework. Direct PEPFAR support of alcohol legislation included the revision of the Alcohol Prevention and Treatment Bill in Namibia and indirect support of an assessment of the Alcohol Levy in Botswana.

In Namibia, the Alcohol Prevention and Treatment Bill was an artifact from South African legislation before Namibia gained independence in 1990, had not been updated since the 1971 Abuse of Dependence-Producing Substances and Rehabilitation Centre Act (Adams, 2015, personal communication). The revision required funding to convene stakeholder groups, including clinicians, legislators, and subject matter experts, and hiring a legal consultant to draft revisions of alcohol-related policies, regulations, and restrictions under the bill. The revision encompassed new alcohol treatment criteria, operational regulations for rehabilitation programs, support groups, and other treatment modalities, and is currently slated for Parliamentary review (Adams, 2015, personal communication).

In Botswana, PEPFAR provided technical assistance to the national government, which in turn commissioned a formal assessment of the Botswana Alcohol Levy by Professor Charles Parry of the South African Medical Research Council (SA MRC) (Parry, 2013). The levy involved a point of sale tax on commercial alcohol set at 30% in 2008 and 40% in 2010. Measuring the impact of the levy proved challenging as no national data of alcohol use existed at the time. Thus, alcohol availability was used as a proxy, measured as local alcohol production plus imports minus exports. This proxy does not account for homebrew, but is considered a fairly reliable measure for commercial alcohol directly affected by the tax. Another proxy for alcohol content analysis used was revenue service data on sales by alcohol type.

The key finding of the assessment was that both proxy measures indicated a decline in alcohol availability and use following the imposition of the levies, from 2008 to 2011. Alcohol availability decreased from 205 000 000 to 180 700 000 L, and alcohol volume by beverage type decreased from 12 500 000 to 8 400 000 L per capita. Per capita consumption was estimated to have decreased from 8 L pure alcohol in 2003–2007 to 7 L in 2010–2012. It is unknown if consumption patterns of non-commercially manufactured (homebrewed) alcohol changed during this time period.

In addition, there were changes over time in secondary measures of alcohol harms following the Botswana alcohol levy. An analysis conducted by the University of Botswana, in collaboration with CDC's National Center for Injury Prevention and Control, found that the overall motor vehicle crash rate was 22% lower in 2010–2011 than the overall crash rate from 2004 to 2009 (Sebego *et al*. 2014). Motor vehicle data were analyzed, finding an increase in total offenses, increase in drivers tested for alcohol use, an increase in proportion of alcohol-related offenses, and a decrease in single vehicle nighttime fatalities from 4.3 per 10 000 in 2007 to 3.3 per 10 000 in 2011, a proxy for alcohol-related motor vehicle fatalities. Significant declines in average fatal crash rates were also observed over this period. Professor Parry noted that findings must be interpreted with caution, as it is difficult to attribute downward trends to the levy alone, since Botswana also implemented stricter drunk driving penalties, increased traffic enforcement, more widespread education on alcohol-related harms, and reduced alcohol trading hours over the same time period. However, the levy was viewed as being highly effective, in concert with these other alcohol-related policies.

## Component 6: Structural interventions

The sixth component of the initiative is the support of structural interventions to reduce opportunities for harmful alcohol use. This encompasses both primary and secondary approaches along the prevention continuum and community and societal level factors within the socio-ecological framework (Fig. 3). Non-legislative structural interventions supported by PEPFAR included a bar-based demonstration project conducted by the International Center for Research for Women (ICRW) to alter informal drinking environments in Namibia in 2012 (Namy *et al*. 2012). This activity took place in the Kabila informal settlement of Katutura, to provide a pilot response to the significant alcohol harms demonstrated in our rapid assessment in this community. Nearly all homes in the Kabila settlement are made of corrugated zinc sheeting, and are without running water and electricity. At the time of the project, there were few tarred roads and most residents live in extreme poverty. In order to quantify the density of alcohol outlets in the area, which measured approximately four square kilometers at the time, the ICRW/PEPFAR team enumerated shebeens, bars, and liquor outlets, totaling 256 in this small location. Given this high density, the project aimed to harness community support and create risk-averse drinking environments as a harm reduction approach. Interventions tested included voluntary reduction in hours of alcohol sales, training servers to limit service to intoxicated patrons and provision of educational materials. Through qualitative and survey approaches, ICRW found that bar owners in Kabila were widely accepting of interventions and found them feasible to implement, and customers found drinking environments safer. Upon assessing drinking patterns before and after intervention implementation, heavy episodic drinking six or more drinks per occasion significantly decreased from 54% to 25%, and this effect in women was correlated to exposure to the intervention. A dose-response relationship was observed with greater reductions in alcohol consumption among persons exposed to longer durations of the interventions (4.1 standard drinks compared to 3.3 standard drinks, *p* < 0.10). Other findings demonstrated reduced sexual risk behavior among ‘heavy’ drinkers (defined as persons who consume two or more drinks per day) exposed to intervention compared with those not exposed, including discussing condoms (87% *v.* 72%), obtaining condoms (93% *v.* 77%) and refusing sex without condoms (62% *v.* 47%) (Namy *et al*. 2012).

As another structural intervention, PEPFAR also supported the creation of the Alcohol Traders Program in Namibia in 2012. This program aimed to promote the rights and responsibilities of formal and informal bar owners as stipulated by the 1998 Liquor Act of Namibia. The PEPFAR team helped design, implement, and evaluate 20 workshops for local bar owners and staff to provide education in the legal parameters of alcohol sales, help establish a Code of Conduct for their businesses, provide safe server and better business guidance, and promote condom distribution in bars.

In Botswana, PEPFAR supported structural interventions in 2012 by providing technical assistance and funding to evaluate HIV surveillance activities in bars in Gaborone. Led by the USAID-funded Project Search: Research to Prevention program at Johns Hopkins University, this surveillance activity surveyed 896 patrons [597 (67%) male, 290 (33%) female] in 60 urban bars. The majority of participants (86%) were ages 20–39 years old, over 40% were educated beyond the secondary school level, 68% were employed, and 75% were unmarried or not cohabiting. Drinking behaviors surveyed found that 47% of the sample overall scored as high-risk drinkers on the AUDIT screen, with AUDIT scores of 8 or above. Heavy episodic drinking six or more drinks in a single occasion during the past week was prevalent among 84% men and 78% women. High-intensity binge drinking 10 or more drinks per occasion was reported by 29% of respondents. Overall, 96% of participants had engaged in sex after drinking alcohol in their lifetime, and 43% had met at least one sexual partner in a bar in the past year. Nearly half (48%) of participants had sex after drinking alcohol with two or more partners in the past year, and 34% of these relationships occurred concurrently. Condom use after the last episode of sex after drinking was reported by 80% of respondents, although 49% reported inconsistent use of condoms after drinking alcohol at any level in the past year. Casual sexual encounters and non-condom use was associated with ‘harmful’ and ‘possible dependence’ drinking levels by AUDIT criteria. The majority of patrons (90%) were aware of sexual risks associated with excessive drinking, but 51% believed their own drinking and sexual behaviors increased risks. A total of 84% of patrons had received an HIV test in their lifetime, and 71% consented for testing at the time of the survey. The weighted HIV prevalence among the survey population was 12.7% overall, 17.7% among women and 10.1% men, respectively. Risk factors for an HIV positive test result included age over 50 years, primary-only education, and divorced marital status. Investigators concluded that there was a high potential for uptake of HIV testing and intervention in bar settings, in the precise context where both sexual and drinking risks take place (Research to Prevention, 2013).

## Component 7: Institutionalize universal prevention messages

The final component of the PEPFAR alcohol initiative was the standardization and refinement of harmful alcohol use prevention messages, an approach that spans the first three areas of the prevention continuum, alcohol availability and use, high-risk drinking, and high-risk sexual behavior, along with the interpersonal and community levels of the socio-ecological framework. PEPFAR supported this component through the design and dissemination of mass media and informational educational and communications (IEC) materials. This activity was the final element to harmonize the various assessments, interventions and activities undertaken, and thus was primarily implemented in Namibia.

**Fig. 4.**
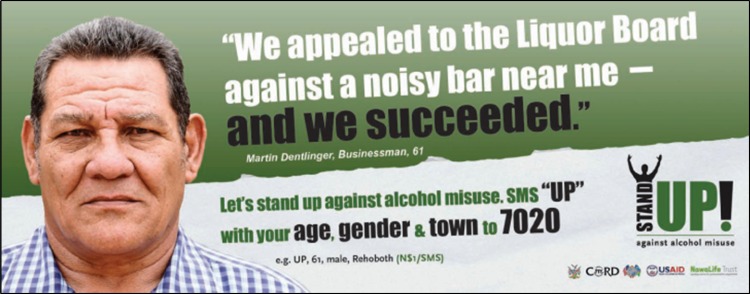
Stand Up Against Alcohol Misuse Campaign, Namibia, 2012 (Nawa LifeTrust, 2014).

PEPFAR supported a local NGO in Namibia, Nawa Life Trust, to develop a mass media and community action campaign called ‘Stand Up Against Alcohol Misuse’ in 2012 (Nawa Life Trust, 2014). This campaign aimed to promote the unacceptability of drunkenness and binge drinking, mobilize communities to take action, and serve as a platform for community members affected or concerned about harmful use of alcohol in their families or areas. The campaign included print, radio and television materials, and an SMS platform for community members to share concerns and request information (Fig. 4).

**Fig. 5.**
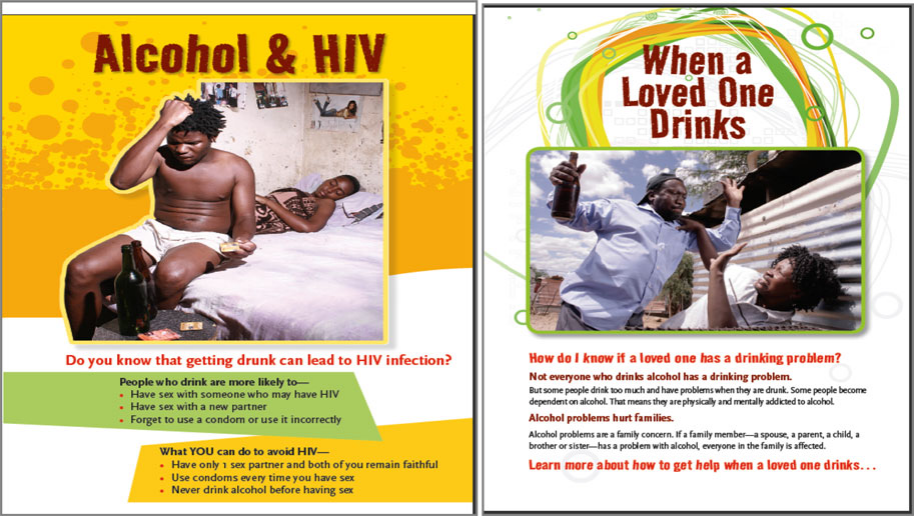
Alcohol Information, Education, Communications Toolkit, Namibia 2012, sample materials.

That same year, the PEPFAR team also designed, evaluated, produced and disseminated the Alcohol Prevention Materials Toolkit. The toolkit was piloted and tested in the several Namibian community settings before finalizing, to best capture local contexts, terms, and issues. The toolkit included a broad range of IEC materials for various audiences, including persons with alcohol use problems, victims of alcohol-related violence, concerned family members, NGOs, healthcare providers, and business owners (Fig. 5). The 13 materials produced were each translated into the three most widely used languages in Namibia: English, Afrikaans, and Oshiwambo. The CORD network headquarters office in Windhoek warehouses and distributes the materials nationwide, including a CD for reprinting the posters, informational booklets and leaflets as needed by recipients.

Through PEPFAR, DOD integrated alcohol messaging in most of the HIV prevention materials currently used by sub-Saharan Africa military communities. Currently under development are two prevention programs that include modules on alcohol based in part on a successful US Marine intervention.

## Discussion

Targeted PEPFAR technical and financial support for alcohol activities resulted in several projects to address the interface between alcohol misuse and HIV prevention, care, and treatment from 2008 to 2013. The seven component objectives of the PEPFAR program are graphically conceptualized along the matrix model depicted in Fig. 3, including examples of programmatic activities implemented in Namibia, Botswana, and other sub-Saharan African settings. This matrix model demonstrates the intersections between primary, secondary, and tertiary alcohol prevention activities along a continuum, with the four interconnected levels of individual, interpersonal, community, and societal factors of the social ecological framework (Fig. 3). Program components 1, 4, and 7 (rapid assessments, network support, and prevention messages, respectively) encompassed the entire scope of the prevention continuum, with activities that aimed to prevent, address, and reduce harmful drinking and associated outcomes. These activities were focused on individual factors (rapid assessments), community factors (network support), and a mix of interpersonal and community-level factors (prevention messages). Other components, such as 5 and 6 (policy and structural interventions), focused on upstream prevention approaches to reduce alcohol availability and harmful use at the societal and community levels, while component 2 (interventions for high-risk populations) addressed secondary prevention approaches to reduce risky drinking and sexual behaviors directly. Finally, component 3 (screening and resources support) aimed to address the adverse health outcomes that result from harmful alcohol use by addressing societal and community factors with downstream approaches.

From the variety of alcohol activities and projects undertaken by PEPFAR, a number of lessons were learned regarding the process, outcome and impact of interventions along this continuum in the resource-limited contexts of Namibia and Botswana. Key successes of the initiative included building upon nascent programs. In Namibia, the Alcohol Unit of the Ministry of Health and Social Services was understaffed, underfunded, and underresourced, but was rich in passion and networks of local champions and organizations committed to curbing harmful alcohol use. This created an ideal platform on which to build a comprehensive, multi-component program. For example, before PEPFAR support, the Namibian CORD network had already organized themselves and cast a wide net to include a multitude of stakeholders ranging from government officials, to clinical providers, to law enforcement. Its core members were committed to addressing alcohol-related harms in Namibia, but lacked capacity and funding to implement public health-focused interventions. Thus, they were ideally suited for both the technical and funding support from PEPFAR and provided the platform for the wide range of PEPFAR-supported alcohol projects. Despite the commitment from government officials there were ongoing challenges with the regulation and enforcement of alcohol sales, due in part to an influential alcohol industry and in part to the popularity of homebrewed alcohol. In Botswana, the most influential anti-alcohol champion was found in the highest office in President Ian Khama (Pitso & Obot, 2011). President Khama introduced the alcohol levy and other alcohol legislation, and enabled the formation of an alcohol unit in the Ministry of Health to use portions of the levy revenue for alcohol programming. With buy-in at such a high level, PEPFAR was able to pilot less conventional alcohol projects in Botswana, such as HIV surveillance in bars, and fill gaps in knowledge regarding local alcohol use patterns and perceptions of interventions.

The platform that PEPFAR was able to build upon in both Namibia and Botswana enabled projects to be piloted in healthcare settings, schools, communities, and alcohol outlets. The breadth of activities generated significant press coverage in both countries and expanded the reach of the projects through public interest, generating over a dozen local media stories. By partnering with media outlets, we were able to help the program extend its reach of core participants and stakeholder networks, and raise substantial awareness in the general public. Demonstration projects were completed with promising results with potential for expansion, such as the ICRW bar-based project in Kabila, Namibia. Other demonstration projects provided the important formative information needed to course-correct before widespread implementation and loss of resources, such as the BMI project in the Namibian Hardap region. The PEPFAR activities also resulted in several key research studies with results that are forthcoming.

Among the many issues in the implementation of the ambitious agenda of PEPFAR alcohol initiative, the primary challenge faced was the complexity of multi-sector, multi-level alcohol programming in the context of varying capacity and limited local resources. It became clear that political will was dynamic concept, particularly in light of arguably more pressing public health concerns in these high HIV burden settings. These moving targets made developing and sustaining a comprehensive alcohol response a more ambitious objective than expected. Broad spectrum alcohol programming spans the sectors of health, injury, mental health, justice, trade, industry, religion, and education. As such, coordination and ‘ownership’ of alcohol programs across sectors can be problematic. As with the tobacco industry, powerful private sector lobbies and significant alcohol revenue generation can influence political decision making. This effect is highly pronounced in developing economies and can undermine the best public health intentions. Continuous political will and endorsement of key gatekeepers, including sustained human and financial resource provision, is essential to build and expand successful alcohol-related research, legislation, and program implementation. A potential structural solution to these challenges could include executive office appointment of a special interagency Alcohol Task Team within the host government, potentially lead by a local alcohol ‘czar.’ This body could champion rapid implementation of activities, coordinate efforts across different departments, and report progress directly to senior executive officials, such as the Office of the President. Such a body structure could enable more streamlined multi-sector engagement while potentially bypassing standard bureaucratic processes that can impede rapid progress. The existence of alcohol teams and champions within the national government structures in both Namibia and Botswana demonstrate the potential for implementation of such an approach in the future.

Challenges in monitoring outcomes of the many projects initiated were also faced through the duration of the initiative. There was no monitoring and evaluation or surveillance system in place to set targets, capture performance indicators, and determine achievements. Creation of these systems was needed for every program piloted, and given the breadth and number of projects, certain activities were left without ideal monitoring components. Whenever possible, we utilized existing resources, such as the AUDIT tool, to measure alcohol use patterns. This may have introduced bias by instituting a 12-month recall parameter for alcohol use indices, and this time period should be reduced in future iterations. Difficulties in evaluating the impact of policy and program interventions were due, in part, to the nearly simultaneous implementation of multiple interventions, making it difficult to determine the individual contribution of each component. If an interagency Alcohol Task Team and czar were to be formally appointed, this governance could require and enable the routinization of monitoring and evaluation systems imbedded in the implemented activities. Although we did not benefit from this Task Team structure and governance in our work, we were able to assess whether changes in consumption of alcohol and selected harms occurred in the context of several interventions, as in the case of the evaluation of the impact of the Botswana alcohol levy. Although another limitation of the Botswana alcohol levy impact is the inability to estimate changes in the consumption of non-commercially manufactured alcohol (homebrew) in relation to the levy implementation, findings of overall decreased consumption are considered robust given the predominance of commercial and sorghum beer consumption in previous investigations.

The most difficult challenge in military settings was found to be garnering approval from military leadership to identify and address alcohol abuse and dependency because of security sensitivity regarding troop readiness. Some of the key lessons learned in developing alcohol abuse programs in African military settings are identifying military healthcare facilities that also serve civilians. By using data that supports both civilian and military personnel, security concerns can be minimized.

There were several research components of the projects implemented that will yield information on the impact of the interventions on behavior change, and results of these activities will be disseminated in the near future. A key lesson learned with special initiative funding is to build core data collection, evaluation and analysis plans into program implementation activities from the outset, to best determine initial outcomes and results. As an underlying principal of the PEPFAR alcohol initiative was to demonstrate and initiate programs and activities, there was limited uptake beyond initial piloting, and sustainability of promising projects was challenging.

Despite these challenges, the application of the matrix model to depict individual, interpersonal, community factors along a continuum of primary, secondary, and tertiary prevention activities could be re-purposed in other settings and for other public health programs. This model could be applied to other prevalent but poorly documented mental health issues in low resource settings, such as depression and suicide, or further explored to more comprehensively address intimate partner violence or child abuse. This model could be further expanded to proactively encompass interconnected systems approach methods and tools, such as systems dynamics modeling and causal loop diagrams, to enable better contextual understanding and feedback, and anticipate and respond to challenges more purposefully and efficiently (Peters, 2014).

Recommendations to reduce alcohol-related morbidity and mortality in low resource settings, particularly regarding sexual risk taking and HIV transmission, echo the actions endorsed by Cherish, Rees and colleagues in 2009 and by the WHO Regional Office for Africa in 2008. The foundational recommendations voiced by these leaders including: (1) raising and increasing political endorsement and community action; (2) conveying clear strategic approaches to measure and mitigate alcohol use as a driver of HIV in strategic plans, national AIDS indicator surveys, and surveillance activities; and (3) developing and implementing structural and programmatic approaches to reduce alcohol availability and harms. These approaches include reducing alcohol availability through taxation, restricting alcohol advertisements and sales, and implementing healthcare screening, care, and treatment services (WHO Regional Office for Africa, 2008; Chersich *et al*. 2009). The matrix model described in this paper, that combines the alcohol prevention continuum with the social ecological model (Fig. 3), is well suited to structure the complexities of these recommendations.

In varying degrees and through various forms of qualitative and quantitative evidence, most of the approaches were trialed and demonstrated through PEPFAR support generally proved feasible and acceptable in the developing settings of Namibia and Botswana. As both of these nations face a heavy dual burden of high HIV prevalence and high rates of heavy episodic alcohol consumption, the importance of addressing both issues in a systematic, comprehensive manner is crucial. The core challenge beyond these foundational approaches is customizing national, international, and regional responses to alcohol-related harms and using local data and champions to direct priorities in context. The final need and challenge of future harmful drinking mitigation endeavors is to plan for long-term sustainability. In settings already heavily affected by alcohol-related social, health, and economic problems, this could best be achieved by instituting and enforcing alcohol control legislation, policies, and regulations; demonstrating the effectiveness of contextually appropriate healthcare, school, and community programs and expanding them broadly; and developing and promoting champions to fuel and invigorate the process.
